# Piquiá Shells (*Caryocar villosum*): A Fruit by-Product with Antioxidant and Antiaging Properties in *Caenorhabditis elegans*

**DOI:** 10.1155/2020/7590707

**Published:** 2020-08-25

**Authors:** Mariana Roxo, Herbenya Peixoto, Pille Wetterauer, Emerson Lima, Michael Wink

**Affiliations:** ^1^Institute of Pharmacy and Molecular Biotechnology, Heidelberg University, Im Neuenheimer Feld 364, 69120 Heidelberg, Germany; ^2^Faculty of Pharmaceutical Sciences, Federal University of Amazonas (UFAM), General Rodrigo 6200, 69077-000 Manaus, Brazil

## Abstract

In a context of rising demand for sustainable antiaging interventions, fruit processing by-products are a promising source of bioactive compounds for the production of antiaging dietary supplements. Piquiá (*Caryocar villosum*) is a native Amazonian fruit consisting of 65% nonedible shells. In the present study, the phytochemical profile of a hydroalcoholic extract of piquiá shells (CV) was characterized by LC-MS/MS analysis. Its antioxidant and antiaging activities were investigated using the nematode *Caenorhabditis elegans* as an *in vivo* model. CV is mainly composed by hydrolysable tannins and triterpenoid saponins. The extract enhanced stress resistance of wild-type and mutant worms by reducing the intracellular levels of reactive oxygen species (ROS) and by increasing their survival against a lethal dose of the prooxidant juglone. These effects involved the upregulation of *sod-3* and downregulation of *gst-4* and *hsp-16.2*, studied through the GFP fluorescent reporter intensity and at the transcriptional level by qRT-PCR analysis. CV extended the lifespan of wild-type worms in a DAF-16/FoxO- and SKN-1/Nrf-dependent manner. Taken together, our findings indicate piquiá shells as potential candidates for nutraceutical applications. Further studies are needed to validate the relevance of our findings to antiaging interventions in humans.

## 1. Introduction

Population aging is leading to an exponential increase in the incidence of age-related health conditions, reflecting extremely high costs for people's welfare and health care systems [1, 2]. This social context has sparked a global demand for antiaging dietary supplements. In recent years, consumers have increasingly preferred sustainable plant-based supplements, shifting the attention of the food, pharmaceutical, and cosmetic industries towards processing of food waste products as an alternative and low-cost source of bioactive compounds with less environmental impact [3]. Within this frame, extracts and compounds isolated from fruit by-products have shown promising potential [4, 5]. Although the antiaging potential of fruit by-products is often ascribed to the potent antioxidant activity displayed by their secondary metabolites (e.g., flavonoids, tannins, and other polyphenols), more detailed *in vivo* studies are needed to support these claims.

Aging results from the complex interplay among genetic, epigenetic, environmental, and stochastic factors [6]. Between the multiple variables involved, oxidative stress has been broadly supported as a considerable contributor to the aging process and thus represents a suitable therapeutic target for the development of novel antiaging interventions [7]. In a more detailed way, as we age, the innate antioxidant network that allows the organism to balance the levels of reactive oxygen species (ROS) from endogenous and exogenous sources is gradually compromised [8]. As a consequence, ROS above physiological levels causes damage to cellular molecules, particularly to proteins, lipids, and DNA (leading to point mutations). The accumulation of oxidative stress-induced damage combined with the decline in repair mechanisms results in mitochondrial dysfunction, loss of proteostasis, and genomic instability, which are shown to contribute to aging itself and to the onset of age-related disorders [9–11]. Therefore, the supplementation of diet with antioxidants, especially those of plant origin, is considered a key strategy for the prevention and symptomatic management of age-related health conditions, such as physical frailty, cancer, metabolic syndrome, cardiovascular disorders and neurodegenerative diseases [12–16].

Model systems have been instrumental in understanding the role of oxidative stress in the aging process and in identifying phytochemical antioxidants with antiaging activity [17–19]. Among the model organisms used for drug discovery in aging research, the roundworm *Caenorhabditis elegans* is considered one of the most cost-effective alternative to mammal testing [20]. Indeed, the *Database of Ageing-related Drugs* (DrugAge) compiles more than 500 compounds with antiaging properties, the majority of which were tested in *C. elegans* [21]. Some of the characteristics that make the worm an ideal platform for aging research are its small size, short life cycle, large offspring, and a lifespan of around three weeks. Moreover, the high genetic tractability and transparency throughout the whole life allow the visualization of anatomical features, fluorescent dyes, and genetically encoded fluorescent markers [22]. Most importantly, *C. elegans* genome is fully sequenced, sharing around 42% orthologues of genes associated with human diseases, and many mutant strains for genes involved in the aging process are readily available [23].

Piquiá (*Caryocar villosum* (Aubl.) Pers.) is a poorly known native Amazonian fruit consisting of 65% nonedible shells (external mesocarp) [24]. The pulp, rich in carotenoids, is the most exploited part of the fruit. It is an important part of the Amazonian diet, usually consumed cooked and used for the production of oil for cooking, cosmetic, and medicinal purposes [25, 26]. The seeds are also edible and a source of oil for cosmetic preparations. Owing to the high oil content of the fruit pulp and seeds, *C. villosum* is often pointed out as a potential crop for biodiesel production [27]. Piquiá shells, rich in tannins and saponins, are traditionally used for the production of ink, hammock dyes, and soap [28]. Previous works on the chemical composition and bioactivity of piquiá were mainly focused on pulp extracts, revealing high phenolic content, *in vitro* ROS scavenging ability, antigenotoxicity, and topical anti-inflammatory activity in rat models [29–32]. To our knowledge, only one study evaluated the bioactive potential of piquiá shells, drawing attention to the presence of polyphenols with strong free-radical scavenging capacity *in vitro* [33]. Since the multiple applications of piquiá pulp generate shells as a by-product, we hypothesized that piquiá shells may represent a sustainable source of antioxidant compounds with potential antiaging properties.

In this study, we characterized the phytochemical composition of a hydroalcoholic extract of piquiá shells (CV) and investigated its antioxidant and antiaging activities in *C. elegans*, additionally exploring underlying mechanisms of action. The extract was evaluated for its effect on the intracellular accumulation of ROS, protection against lethal oxidative stress, gene expression, and lifespan extension of wild-type and mutant worms for genes involved in stress resistance and longevity in *C. elegans*. CV-mediated changes in gene expression were studied using transgenic strains carrying a GFP reporter for the gene of interest and qRT-PCR analysis.

## 2. Material and Methods

### 2.1. Plant Material and Extraction Procedure

Ripe fruits of *Caryocar villosum* were collected at the Amazonian Ducke Forest Reserve, Manaus, Brazil. A hydroalcoholic extraction (8 : 2 ethanol/water) of the shells was performed at room temperature. The final extract of *Caryocar villosum* fruit shells was concentrated by rotary evaporation and spray dried, yielding a dark yellow fine powder. For all the experiments, the extract was freshly diluted in sterile water and insoluble particles removed by centrifugation (10,000 rpm for 5 min at 4°C).

### 2.2. Phytochemical Characterization of the Extract—LC-MS/MS Analysis

LC-MS/MS analysis was performed on a Finnigan LCQ-Duo ion trap mass spectrometer with an ESI source (ThermoQuest, Hyland Scientific, Stanwood, WA, USA), coupled to a Thermo Scientific Accela HPLC system (MS pump plus, autosampler, and PDA detector plus) with an EC 150/3 Nucleodur 100-3 C18ec column (Macherey-Nagel, Dueren, Germany). A gradient of water and acetonitrile (ACN) with 0.1% formic acid each was applied at 30°C as follows: 0–15% ACN in 15 min; 15% ACN for 10 min; 15–35% ACN in 20 min; 35% ACN for 10 min; 35–50% ACN in 20 min; 50–100% ACN in 10 min; and 100% ACN for 15 min. The flow rate was 0.5 mL/min. The injection volume was 20 *μ*L. All samples were measured in the positive and negative mode. The MS was operated with a capillary voltage of 10 V, source temperature of 240°C, and high purity nitrogen as a sheath and auxiliary gas at a flow rate of 80 and 40 (arbitrary units), respectively. The ions were detected in a mass range of 50-2000 *m*/*z*. Collision energy of 35% was used in MS/MS for fragmentation. Data acquisitions and analyses were executed by the Xcalibur™ 2.0.7 software (Thermo Scientific, Waltham, MA, USA).

### 2.3. *In Vitro* Antioxidant Activity

The *in vitro* antioxidant activity of CV was characterized through the standard assays DPPH (1,1-diphenyl-2-picrydrazyl) and TEAC (trolox equivalent antioxidant capacity) assessed through ABTS (2,2'-azino-bis(3-ethylbenzothiazoline-6-sulfonic acid) decolorization, performed as described in our previous study [34]. Vitamin C (AppliChem GmbH, Darmstadt, Germany) and gallic acid (Sigma-Aldrich GmbH, Steinheim, Germany) were used as positive controls.

### 2.4. *In Vivo* Antioxidant Activity

#### 2.4.1. *C. elegans* Strains and Maintenance

All strains used in this study were provided by the Caenorhabditis Genetics Center (CGC, University of Minnesota, Minneapolis, MN, USA), namely **N2** (WT), **CF1038** [daf-16(mu86)], **EU1** [skn-1(zu67)], **VC475** [hsp-16.2(gk249)], **CF1553** [(pAD76) sod-3p::GFP + rol-6(su1006)], **CL2166** [(pAF15)gst-4p::GFP::NLS], **TJ375** [hsp-16.2p::GFP], **TJ356** [daf-16p::daf-16a/b::GFP + rol-6(su1006)], **LD1** [skn-1b/c::GFP + rol-6(su1006)], and *Escherichia coli ***OP50** (uracil auxotroph).

The worms were cultured on solid nematode growth medium (NGM), fed on living *E. coli* OP50, and maintained at 20°C [35]. To obtain synchronized populations, the eggs were isolated using a combination of bleaching and sucrose density separation methods and allowed to hatch overnight in M9 buffer [36]. For the experiments, freshly hatched L1 worms were transferred to liquid S-medium inoculated with living *E. coli* OP50 (OD_600_ = 1.0) and treated according to the correspondent assay, in a final volume of 2 mL/petri dish (35 × 10 mm). All assays were performed at 20°C.

#### 2.4.2. Fluorescence Microscopy and Image Analysis

To analyze the effect of different treatments on the expression of the fluorescent reporter GFP (green fluorescent protein) and the levels of DCF (2',7'-dichlorofluorescein), living worms were mounted onto a glass slide with a drop of 10 mM sodium azide (AppliChem GmbH, Darmstadt, Germany). Fluorescence images (*λ*_ex_ 488 nm and *λ*_em_ 540) of at least 30 worms/treatment were acquired at constant exposure time for each experiment, using a Keyence Biorevo BZ-9000 fluorescence microscope (Keyence Deutschland GmbH, Neu-Isenburg, Germany). The fluorescence intensity was densitometrically determined in mean pixels using ImageJ version 1.52 (National Institutes of Health, Bethesda, MD, USA), an open source image processing and analysis software.

#### 2.4.3. Measurement of Intracellular ROS Levels by DCF Assay

N2, CF1038, EU1, and VC475 worms at L1 larval stage were treated with different concentrations of CV (50, 100, and 200 *μ*g/mL), excluding the control group. A positive control of EGCG was included (25 *μ*g/mL or 50 *μ*g/mL). On the first day of adulthood, the worms were incubated for 1 hour at 20°C with 20 *μ*M DCF-DA (2',7'-dichlorofluorescein diacetate) (Sigma-Aldrich GmbH, Steinheim, Germany), subsequently washed with M9 buffer to remove residual dye, and analyzed by fluorescence microscopy [37]. ROS levels are expressed as percentage of control (mean ± SEM) and calculated from the mean pixel intensity of the worm's whole body.

#### 2.4.4. Survival Assay under Juglone-Induced Oxidative Stress

N2, CF1038, EU1, and VC475 worms at L1 larval stage were treated as previously described. On the first day of adulthood, approximately 80 worms per treatment were transferred onto new plates containing freshly inoculated S-medium and 80 *μ*M juglone (Sigma-Aldrich GmbH, Steinheim, Germany) [38]. After 24 h, the survivors were counted. Worms with no visible response to a gentle touch stimulus were scored dead. Survival rate is expressed as percentage (mean ± SEM).

#### 2.4.5. Expression of Stress Resistance-Related Genes

*(1) Subcellular Localization of DAF-16 and SKN-1*. TJ356 and LD1 transgenic worms at L4 stage were treated for exactly one hour prior fluorescence microscopy imaging. Each worm was then categorized according to the GFP pattern of expression observed (cytosolic, intermediate, or nuclear) as previously described by our group [39].

*(2) Quantification of sod-3, gst-4, and hsp-16.2 Expression under Basal Stress Conditions and/or Juglone-Induced Oxidative Stress*. CF1553, CL2166, and TJ375 transgenic worms were treated at L1 stage. After 48 h of treatment, the adult worms were exposed or not to 20 *μ*M of juglone for 24 h and then analyzed by fluorescence microscopy [37, 40]. Results are shown as percentage of control (mean ± SEM) calculated from the mean pixel intensity of the worm's tail (*sod-3*), whole body (*gst-4*), or head (*hsp-16.2*).

*(3) Quantification of Relative sod-3, gst-4, and hsp-16.2 mRNA Levels: RNA Purification and qRT-PCR*. N2 worms at L1 stage were treated as previously mentioned. After 72 h, the worms were collected by gravity, washed three times in M9 buffer to remove residual *E. coli* OP50, and flash frozen in liquid nitrogen. Total RNA was isolated and purified using the FastGene RNA Premium Purification Kit (Nippon Genetics Europe GmbH, Dueren, Germany). RNA purity and concentration were assessed by spectrophotometry at 260 and 280 nm. Purity and integrity were further confirmed on an agarose gel. cDNA was synthesized from 1 *μ*g of pure RNA using the Nippon FastGene Scriptase II kit. qRT-PCR was performed using PCR Biosystems SyGreen Mix (PCR Biosystems Inc., Wayne, PA, USA) on a Roche LightCycler 96 System (Roche Diagnostics Deutschland GmbH, Mannheim, Germany). Results are presented as fold relative mRNA levels (2^-*ΔΔ*Ct^), calculated from the *ΔΔ*Ct values normalized to *cdc-42* as a reference gene. The sequences of the PCR primers used were previously published [41].

### 2.5. Lifespan

Synchronized N2, CF1038, and EU1 worms were grown in inoculated S-medium until the first day of adulthood. The worms were then separated into treatment groups of approximately 100 individuals. During the egg-laying period, the worms were transferred every day to freshly inoculated liquid medium with extract and posteriorly every second day. Worms with internally hatched progeny or extruded gonads were censored. Worms no longer responding to a gentle touch with a platinum wire were scored as dead and excluded from the plates. The results are expressed as percent survival.

### 2.6. Feeding Behavior, Development, and Reproduction

#### 2.6.1. *E. coli* OP50 Growth and Food Intake

The effect of CV treatment on *E. coli* OP50 growth and food intake of wild-type worms was assessed through the changes in absorbance at 600 nm [42]. Briefly, 200 *μ*L of freshly *E. coli* OP50 inoculated S-medium (OD_600_ = 1) per well was incubated at 20°C with or without 200 *μ*g/mL CV on a 96-well plate. Measurements were taken every 24 h for a period of 72 h using a microplate reader (Tecan, Männedorf, Switzerland). Results are presented as fold *E. coli* OP50 growth normalized to the absorbance of the respective treatment at day 0 (mean ± SEM). For the food intake assays, approximately 20 worms (N2 at L1 stage) were added to the inoculated S-medium with or without 200 *μ*g/mL CV, in a final volume of 200 *μ*L/well. Due to the observed oxidation of the plant extract, wells containing noninoculated S-medium with or without plant extract were used as blank. Measurements followed as described. Results are presented as fold food intake normalized to the absorbance at day 0 (mean ± SEM).

#### 2.6.2. Body Area

The body area of N2 worms treated from L1 stage with CV was determined using the worms imaged for DCF assay (Section 2.4.3). Results are expressed as percentage of control (mean ± SEM).

#### 2.6.3. Brood Size

Synchronized N2 worms at L4 larval stage were placed one by one on individual NGM agar plates. An *E. coli* OP50 lawn was provided as food source. In the treatment groups, the plant extract was added to the bacterial lawn in a concentration of 200 *μ*g/mL. The worms were transferred every day to fresh medium, and after 24 h, the eggs were counted for 5 d using a dissecting microscope. Brood size results are expressed as number of eggs laid per worm each day (mean ± SEM).

### 2.7. Statistical Analyses and Data Interpretation

All graphs were performed using Prism 8 for macOS version 8.2.1 (GraphPad Software, San Diego, CA, USA). For all assays except lifespan, the statistical differences between groups were calculated using one- or two-way ANOVA (brood size assay) followed by Tukey's multiple comparisons test using GraphPad Prism. Lifespan assays were analyzed by Kaplan-Meier followed by the log-rank test using IBM SPSS Statistics for Macintosh version 25.0 (IBM Corp., Armonk, NY, USA).

## 3. Results and Discussion

### 3.1. Phytochemical Composition of CV

**Figure 1:**
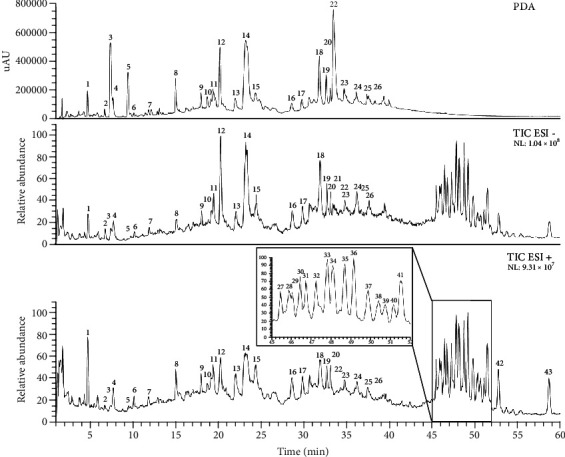
Chromatogram (PDA) and total ion current (TIC) of the hydroalcoholic extract of *Caryocar villosum* fruit shells obtained by LC-MS/MS in negative (ESI -) and positive (ESI +) mode. Peak numbers correspond to the tentatively identified compounds presented in Tables 1 and 2 (NL: normative level).

The phytochemical composition of CV was analyzed by LC-MS/MS (Figure 1). A total of 43 peaks were characterized and 33 compounds were tentatively identified by their molecular mass, fragmentation pattern, and absorption based on literature data (Tables 1 and 2). Overall, CV contains polyphenols belonging to the major group of the hydrolysable tannins (including ellagi- and gallotannins) and triterpenoid saponins. Phenolic compounds showed strong absorption and were better characterized in the negative mode, while for saponins, no absorption was detected, and the positive ion mode provided more detailed structural information.

Gallic acid, ellagic acid, and respective derivatives were previously reported as major phenolic compounds of *C. villosum* extracts from both shells and pulp [33, 43]. Peaks **2–5**, **12**, **18**, and **22** correspond to compounds already reported for *C. villosum*. To our knowledge, the tentatively identified hydrolysable tannins corresponding to the peaks **1**, **8**, **9**, **13**, **14**, **15**, **16**, **17**, **19**, **20**, **23**, and **26** are reported here for the first time in *Caryocar* spp. Peak **1** with *m*/*z* 355 [M-H^+^]^−^, and the fragment ion with *m/z* 337 [M-H^+^-H_2_O]^−^ was tentatively identified as chebulic acid [44]. Peak **8** with *m*/*z* 469 [M-H^+^]^−^ produced the fragment ion with *m*/*z* 425 [M-H^+^-CO_2_]^−^ as described by Wyrepkowski et al. [45] for valoneic acid dilactone. Peaks **9** and **15** probably correspond to galloyl-valoneoyl-glucoside isomers. In the negative mode, they show a *m*/*z* of 801 [M-H^+^]^−^ with the major fragment ion with *m*/*z* 757 [M-H^+^-CO_2_]^−^. This ion gives further fragment ions with *m*/*z* 633, 613, and 301 as reported previously for galloyl-decarboxy-valeoyl-glucoside [46]. Peaks **13**, **20**, and **26**, exhibiting the same quasimolecular ion with *m*/*z* 951 [M-H^+^]^−^, same *λ*_max_ at 171 nm, and same fragmentation pattern, seem to be isomers of the same substance. Due to the major fragment ions with *m*/*z* 907 [M-H^+^-CO_2_]^−^ and 301, a typical MS/MS fragment produced after lactonization of the hexahydroxydiphenoyl (HHDP) group into ellagic acid, they were tentatively assigned to HHDP-valoneoyl-glucoside, as proposed by Yisimayili et al. [47]. According to the same authors, the peak **14**, also with *m*/*z* 951 [M-H^+^]^−^ but different absorption and fragmentation, was identified as galloyl-HHDP-DHHDP-hexoside. Peak **23** has with *m*/*z* 907 [M-H^+^]^−^ the same ion mass as the major fragment of HHDP-valoneoyl-glucoside, and also, its fragmentation pattern is very similar. Therefore, this compound was tentatively identified as HHDP-decarboxy-valoneoyl-glucoside. Peak **16** with *m*/*z* 925 [M-H^+^]^−^ and a major fragment ion with *m*/*z* 301 show the same fragmentation pattern as described for phyllanthusiin C by Catarino et al. [48]. Peak **17** with *m*/*z* 969 [M-H^+^]^−^ and major fragment ions with *m*/*z* 925 [M-H^+^-CO_2_]^−^, 633, and 301 were tentatively identified as phyllanthusiin B by comparison with the same authors. Peak **19** has a quasimolecular ion at *m*/*z* 785 [M-H^+^]^−^, a major fragment ion with *m*/*z* 301 and another typical fragment ion with *m*/*z* 633 [M-H^+^-galloyl]^−^. According to Taamalli et al. [49] and Yisimayili et al. [47], this fragmentation pattern corresponds to digalloyl-HHDP-glucose.

The tentatively identified triterpenoid saponins have been already described for stem bark and fruits of *C. villosum* and *C. glabrum* [50–52]. The source fragmentation data in Table 2 corresponds to the previously reported fragmentation patterns.

### 3.2. *In Vitro* Antioxidant Activity

DPPH and ABTS assays were performed to characterize the *in vitro* antioxidant activity of CV (Table 3). The extract showed a strong radical scavenging activity, comparable to that of ascorbic acid, a well-known potent antioxidant used as a positive control in our study. Gallic acid, previously reported as a major compound of *C. villosum* extracts and also identified in the extract of the present study, revealed the highest radical scavenging capacity in both assays. These results, although without direct biological significance, may serve as comparative values for further studies on the antioxidant activity of *Caryocar* spp. extracts.

### 3.3. *In Vivo* Antioxidant Activity—*C. elegans*

DAF-16/FoxO and SKN-1/Nrf are key transcription factors in the regulation of stress resistance, protein homeostasis, and lifespan in *C. elegans* [55]. The activity of these transcription factors is controlled in parallel but independently by the insulin/IGF-1 pathway (IIS), an evolutionarily conserved pathway that regulates aging from worms to mammals. Under favorable conditions, DAF-16 and SKN-1 are inhibited by the same IIS kinases (SGK-1 and AKT-1/2) through phosphorylation and cytoplasmic retention [19, 56]. Upon different types of stress, reduced IIS signaling releases the inhibition leading to nuclear translocation and consequent transcriptional activation or suppression of stress-response genes. Among others, DAF-16 directly regulates the transcription of *sod-3*, coding for the phase I antioxidant enzyme superoxide dismutase 3 (SOD-3), and coregulates with the heat-shock factor 1 (HSF-1) the expression of *hsp-16.2*, coding for the heat-shock protein 16.2 (HSP-16.2) [57, 58]. SKN-1 is mainly involved in the regulation of phase II detoxification genes, such as *gst-4*, coding for glutathione*S*-transferase 4 (GST-4) [56].

In the present study, we initially tested the antioxidant activity of CV by evaluating its effect on the *in vivo* scavenging of ROS and resistance against lethal oxidative stress. The mechanisms of action involved in the antioxidant activity of CV were further explored by analyzing the effect of the extract on the activation of DAF-16 and SKN-1 and respective downstream targets—*sod-3*, *gst-4*, and *hsp-16.2*.

#### 3.3.1. ROS Levels and Survival against Juglone-Induced Oxidative Stress

**Figure 2:**
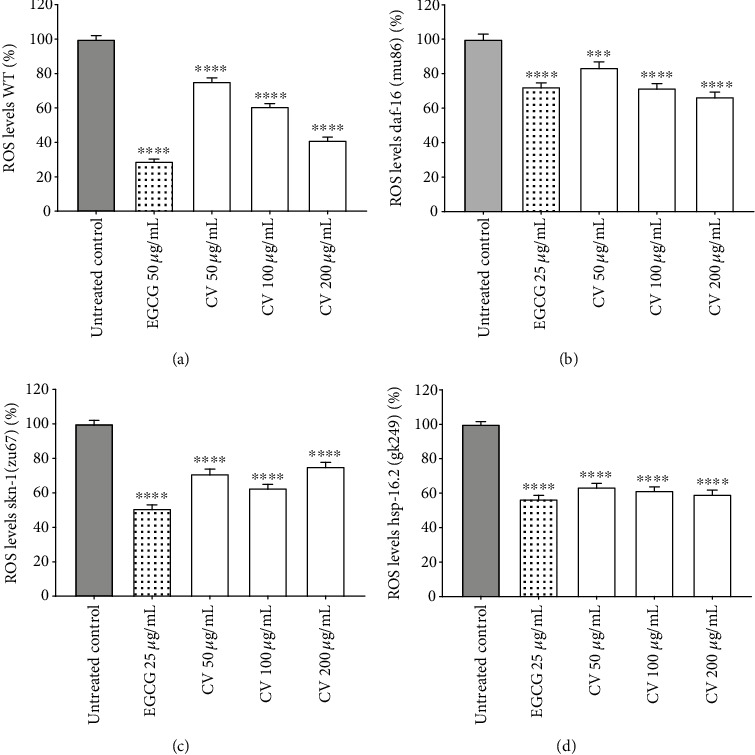
Intracellular ROS accumulation under basal stress conditions of wild-type (a) and mutant worms for *daf-16* (b), *skn-1* (c), and *hsp-16.2* (d) treated with *Caryocar villosum* fruit shells (CV). The results are expressed as mean percentage of control ± SEM from at least three independent experiments, calculated from DCF intensity of the worm's whole body (∗∗∗∗*p* < 0.0001, compared to the untreated control).

In a first set of experiments, we assessed the effect of CV extract on the basal levels of ROS in wild-type worms (N2) using the oxidation-sensitive fluorescent probe DCF [59]. The extract was able to significantly reduce the intracellular ROS accumulation at all concentrations tested in a dose-dependent manner (Figure 2(a)). At the maximum concentration tested (200 *μ*g/mL), CV reduced the levels of ROS by 58.74 ± 1.76% in comparison to the untreated control (*p* < 0.0001).

To elucidate the role of DAF-16 and SKN-1 in the observed activity, knock-out mutants for these transcription factors were employed (CF1038 and EU1, respectively). Although to a lesser extent, CV also significantly reduced the levels of ROS in both mutants (Figures 2(b) and 2(c)). For the *skn-1* mutant, CV displays a U-shaped dose-response curve, with the highest ROS scavenging activity at 100 *μ*g/mL. This result might be indicative of a prooxidant effect of the extract at higher doses as a direct consequence of the absence of important detoxification genes. Additionally, in the *hsp-16.2* mutant (VC475), CV decreased the accumulation of ROS with slight differences between concentrations tested (Figure 2(d)). Representative fluorescence images of the living worms analyzed for the quantification of ROS can be found in the supplementary information (Figure S1).

Yamaguchi et al. [33] also demonstrated the ability of a piquiá shells extract to scavenge free radicals *in vitro*. In a cell-based antioxidant assay, the extract was able to inhibit oxidation reducing by almost 100% the ROS levels, and in a model of LPS-stimulated macrophages, it nearly suppressed the production of nitric oxide, a free radical that acts as proinflammatory mediator.

**Figure 3:**
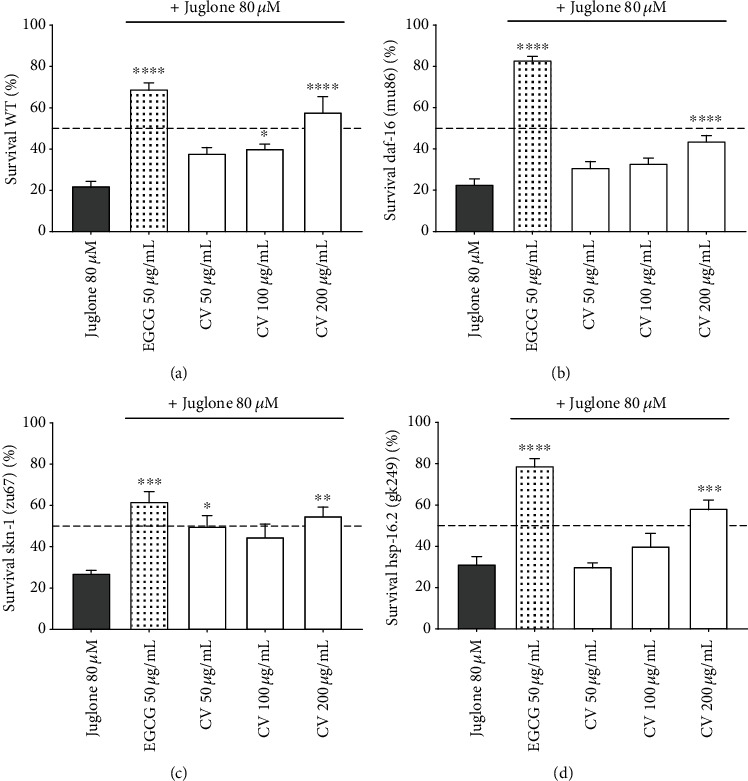
Effect of *Caryocar villosum* fruit shells (CV) on the survival rate of wild-type (N2) and mutant worms for *daf-16* (b), *skn-1* (c), and *hsp-16.2* (d), exposed to 80 *μ*M juglone to induce lethal oxidative stress. Survival rate is expressed as mean percentage survival ± SEM of at least four independent experiments (∗*p* < 0.05, ∗∗*p* < 0.01, and ∗∗∗*p* < 0.001, compared to juglone).

In order to investigate whether CV confers resistance against acute oxidative stress, survival assays under a lethal dose of the prooxidant juglone were performed using the strains previously tested. Juglone, a naphthoquinone isolated from walnut tree (*Juglans regia* L.), is commonly used as an oxidative stress inducer in *C. elegans* [60, 61]. Depending on the concentration applied, it can induce mild to lethal oxidative stress via a redox cycling mechanism. Based on previous investigations carried out in our laboratory, we selected a dose of 80 *μ*M juglone to induce lethal oxidative stress [62]. Wild-type worms treated with 200 *μ*g/mL CV were the most resistant to 80 *μ*M juglone (Figure 3(a)), with a survival rate of 57 ± 7.49% in comparison to 22.20 ± 2.14% in the juglone group (*p* < 0.0001). As expected, the *daf-16* mutant, which previously exhibited higher levels of ROS, showed higher sensitivity to oxidative stress (Figure 3(b)). For the *skn-1* mutant, the highest survival rate corresponded to the treatment group with the highest levels of ROS (Figure 3(c)). This result shows that the mild prooxidant effect of CV at 200 *μ*g/mL positively mediated the response of *skn-1* mutants to lethal oxidative stress, suggesting a hormetic dose-response [63, 64]. Similar results were reported for other tannin-rich plant extract [65] and several dietary antioxidants, such as caffeine and related methylxanthines [41], tomatidin [66], and quercetin [67]. Mutant worms for *hsp-16.2* showed increased resistance to oxidative stress only when treated with 200 *μ*g/mL CV (Figure 3(d)). Taken together, our results suggest that the enhanced stress resistance elicited by CV treatment is, apparently, not strictly dependent on *daf-16*, *skn-1*, and *hsp-16.2*. However, if taken quantitatively, these results might also allow us to conclude that the absence of DAF-16 signaling negatively impacts the antioxidant capacity of CV, since the *daf-16* mutant showed overall higher ROS levels and lower survival rates in comparison to the wild-type worms.

#### 3.3.2. Expression of Stress Resistance-Related Genes

**Figure 4:**
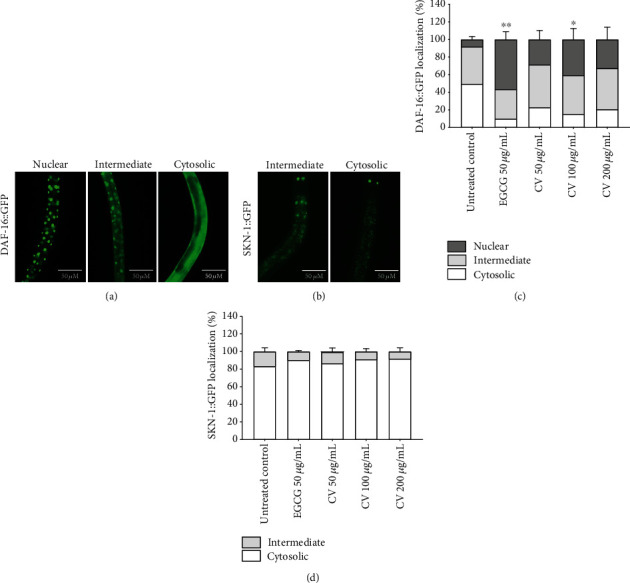
Effect of *Caryocar villosum* fruit shells (CV) on the intracellular localization of DAF-16 and SKN-1. (a) Representative images of TJ356 worms with nuclear, intermediate, and cytosolic DAF-16::GFP localization. (b) Representative images of LD1 worms with intermediate and cytosolic SKN-1::GFP localization. (c) Intracellular localization of DAF-16. (d) Intracellular localization of SKN-1. The results are presented as mean percentage of worms showing each pattern of localization of at least three independent experiments. For DAF-16::GFP, error bars show the mean percentage of worms exhibiting nuclear localization ± SEM, and the statistical differences also refer to this pattern of localization (∗*p* < 0.05, ∗∗*p* < 0.01, compared to the untreated control). For SKN-1::GFP, error bars show the mean percentage of worms exhibiting intermediate localization ± SEM.

Plant extracts rich in polyphenols were previously found to exert antioxidant and lifespan-promoting effects in *C. elegans* by inducing the transcriptional activity of DAF-16 and SKN-1, thus modulating the expression of genes related to stress resistance and longevity [37, 68, 69]. To clarify which genes are modulated by CV to enhance oxidative stress resistance in *C. elegans*, we initially analyzed its effect on the activation of DAF-16 and SKN-1. The effect of different concentrations of CV on the subcellular localization patterns of both transcription factors was determined using the transgenic strains TJ356 and LD1 carrying a translational GFP reporter for DAF-16 and SKN-1, respectively (Figures 4(a) and 4(b)). CV treatment induced the translocation of DAF-16 to the nucleus (Figure 4(c)) but failed to activate SKN-1 (Figure 4(d)). DAF-16 nuclear localization was induced by 100 *μ*g/mL in 32.51 ± 12.40% of the worms, in comparison to the untreated control (*p* < 0.05).

The expression levels of DAF-16 and SKN-1 target genes, *sod-3*, *gst-4*, and *hsp-16.2*, were subsequently determined using strains carrying a GFP reporter coupled to the promotor of the respective gene and through qRT-PCR analysis. Initially, we employed the transgenic strain CF1535 to investigate the expression of *sod-3*::GFP under basal stress conditions and under mild oxidative stress, induced by a dose of 20 *μ*M juglone. This concentration of juglone was previously shown to induce the expression of stress resistance-related genes without compromising the vital functions of the worms [62, 70].

**Figure 5:**
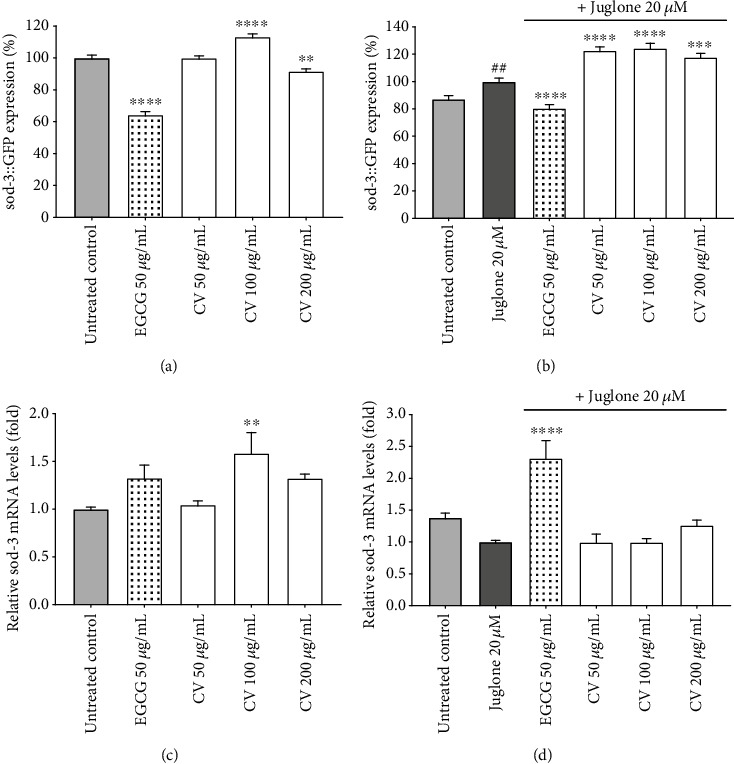
Effect of *Caryocar villosum* fruit shells (CV) on superoxide dismutase 3 (*sod-3*) expression under basal and mild oxidative stress, induced by a dose of 20 *μ*M juglone. (a) *sod-3*::GFP expression levels under basal stress conditions. (b) *sod-3*::GFP expression levels under juglone-induced oxidative stress. (c) Relative *sod-3* mRNA levels of wild-type worms under basal stress conditions. (d) Relative *sod-3* mRNA levels of wild-type worms under 20 *μ*M juglone-induced oxidative stress. Data are representative of three independent experiments. GFP expression levels are expressed as mean percentage of untreated control (basal stress) or juglone (induced stress) ± SEM (∗∗*p* < 0.01, ∗∗∗*p* < 0.001, and ∗∗∗∗*p* < 0.0001, compared to the respective control) (^##^*p* < 0.01, compared to the untreated control). Relative mRNA levels are presented as mean fold relative mRNA levels, calculated from *ΔΔ*Ct values normalized to *cdc-42* as a reference gene (∗∗*p* < 0.01, ∗∗∗∗*p* < 0.0001, compared to juglone).

Representative fluorescence images of the living worms analyzed for the quantification of *sod-3* can be found in the supplementary information (Figures S2 and S3). Under basal stress conditions, 100 *μ*g/mL CV significantly increased the fluorescence intensity by 13.2 ± 1.83%, in comparison to the untreated control (*p* < 0.0001) (Figure 5(a)). In contrast, at 200 *μ*g/mL, we could observe a significant decrease in GFP intensity. A mild dose of 20 *μ*M juglone resulted in a more pronounced induction of *sod-3*::GFP at all CV concentrations tested (Figure 5(b)). A maximum upregulation of 24.3 ± 3.74% occurred at 100 *μ*g/mL, in comparison to the juglone treatment (*p* < 0.0001).

The expression of *sod-3* was also investigated at the transcriptional level through qRT-PCR analysis. The mRNA levels of *sod-3* under basal stress conditions followed the same pattern as the GFP intensity (Figure 5(c)). As expected, in worms treated with CV 100 *μ*g/mL, the relative mRNA levels were higher than in the untreated control. This result, combined with the GFP intensity and the DAF-16 localization pattern previously observed, suggests that at 100 *μ*g/mL CV induces the translocation of DAF-16 to the nucleus resulting in upregulation of *sod-3*, its target gene.

Treatment with 200 *μ*g/mL CV resulted in a maximum reduction of ROS levels by 58.74 ± 1.76% (Figure 2(a)); however, at this concentration, the expression levels of *sod-3* were reduced. This result is apparently contradictory, because low levels of ROS are usually correlated with higher expression levels of the antioxidant enzyme *sod-3* [37, 71]. Despite that, a similar effect was also reported for other phytochemical and synthetic antioxidants. Quercetin and resveratrol were shown to protect *C. elegans* from oxidative stress and to reduce the intracellular levels of ROS, although both compounds reduced the expression of *sod-3* [72, 73]. The standard antioxidants ascorbic acid, butylated hydroxyanisole (BHA), and sodium ascorbate were also reported to reduce ROS levels in *C. elegans* simultaneously reducing the expression of *sod-3* [74]. According to the authors, the sharp reduction in the levels of ROS induced by potent antioxidants leads to the absence of cellular oxidative burden, resulting in the repression of *sod-3* expression. Most probably, this argument explains the results obtained in this study for 200 *μ*g/mL CV. Our findings suggest that 200 *μ*g/mL is the threshold antioxidant concentration from which CV ceases to induce the expression of *sod-3*.

Under oxidative stress, an inverse correlation between mRNA levels and *sod-3*::GFP intensity was observed (Figure 5(d)). The relative levels of mRNA were measured in wild-type worms, and the GFP intensity was determined using a mutant strain carrying a transcriptional reporter. In *C. elegans* research, the transcriptional fusion approach is often used alternatively to qRT-PCR to investigate changes in gene expression. However, transcriptional reporters may lack potential regulatory elements located beyond the 5′ end or at the 3 ′-unstranslated region, resulting in misleading patterns of gene expression [75]. Reasoning from this fact, the effect of CV treatment on the expression of *sod-3* might be better represented by the qRT-PCR data rather than the GFP intensity. In any case, these results deserve further attention.

**Figure 6:**
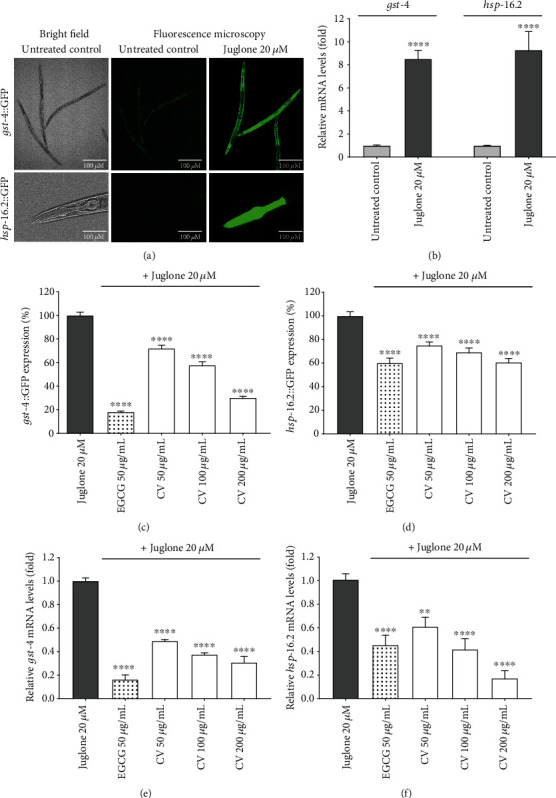
Effect of *Caryocar villosum* fruit shells (CV) on heat-shock protein 16.2 (*hsp-16.2*) and glutathione *S*-transferase 4 (*gst-4*) expression under mild oxidative stress, induced by a dose of 20 *μ*M juglone. (a) Representative images of CL2166 (*gst-4*::GFP) and TJ375 (*hsp-16.2*::GFP) worms. (b) Relative mRNA levels of *gst-4* and *hsp-16.2* of untreated and juglone treated wild-type worms. (c) *gst-4*::GFP expression levels. (d) *hsp-16.2*::GFP expression levels. (e) Relative *gst-4* mRNA levels of wild-type worms. (f) Relative *hsp-16.2* mRNA levels of wild-type worms. Data are representative of three independent experiments. GFP expression levels are expressed as mean percentage of juglone ± SEM (∗∗∗∗*p* < 0.001, compared to juglone). Relative mRNA levels are presented as mean fold relative mRNA levels in comparison to the juglone treatment ± SEM, normalized to *cdc-42* as a reference gene (∗∗*p* < 0.01, ∗∗∗∗*p* < 0.001, compared to juglone).

Considering that *gst-4* and *hsp-16.2* are stress inducible genes, we tested the effect of CV on the expression levels of these genes only under mild oxidative stress. A dose of 20 *μ*M juglone was previously reported by our and other research groups as a mild oxidative stress stimulus, which is able to strongly induce the expression of *gst-4*::GFP and *hsp-16.2*::GFP [37, 39, 70, 73]. Accordingly, in this study, worms under juglone-induced stress showed strong GFP intensity, which was almost undetectable in the untreated worms (Figure 6(a)). CV treatment reduced the fluorescence intensity of both *gst-4*::GFP (CL2166) and *hsp-16.2*::GFP (TJ375) in a dose-dependent manner (Figures 6(c) and 6(d)). At 200 *μ*g/mL, CV reduced the expression of *gst-4*::GFP by 69.95 ± 1.31% and of *hsp-16.2*::GFP by 39.40 ± 3.37%, in comparison to the juglone control (*p* < 0.0001). Representative fluorescence images of the living worms analyzed for the quantification of *gst-4* and *hsp-16.2* can be found in the supplementary information (Figures S4 and S5).

Juglone also increased the expression of *gst-4* and *hsp-16.2* in wild-type worms at the transcriptional level. The expression of *gst-4* and *hsp-16.2* increased 8.5-fold and 9.3-fold, respectively, in comparison to the untreated control (Figure 6(b)). For both genes, the GFP intensity was consistent with the relative mRNA expression levels (Figures 6(e) and 6(f)**)**. In worms subjected to the same dose of juglone and 200 *μ*g/mL CV, *gst-4* and *hsp-16.2* expression decreased to 0.33 and 0.17-fold, respectively, in comparison to the juglone treatment (*p* < 0.0001).

GST-4 is a phase II detoxification enzyme that catalyzes the conjugation of several exogenous and endogenous hydrophobic electrophiles with the reduced form of glutathione, resulting in the detoxification of ROS and xenobiotic compounds [76]. HSP-16.2 acts as a molecular chaperone, binding to partially denatured proteins to avoid protein aggregation. The capacity of dietary antioxidants to suppress the stress-induced expression of genes coding for these proteins is widely interpreted as proof of their ability to reduce oxidative stress in *C. elegans* [38, 77–79]. Accordingly, the downregulation of *gst-4* and *hsp-16.2* elicited by CV further confirms the extract's ability to protect *C. elegans* from oxidative stress and, presumably, from the damage it may induce.

#### 3.3.3. Lifespan Extension

**Figure 7:**
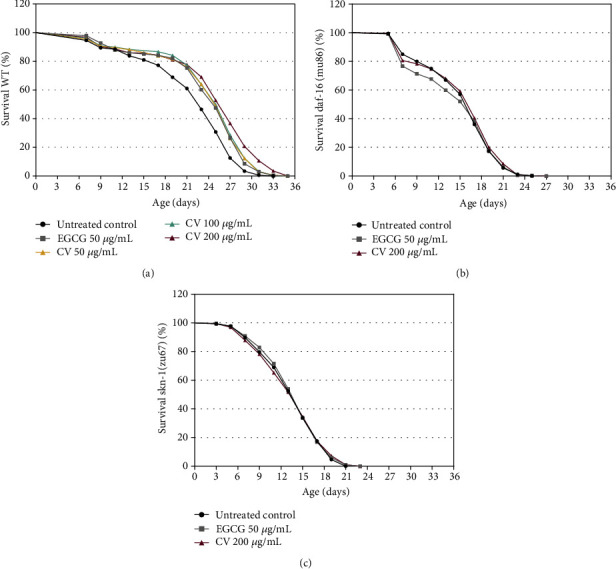
Lifespan of wild-type (a) and mutant worms for *daf-16* (b) and *skn-1* (c) treated with *Caryocar villosum* fruit shells (CV). Lifespan is expressed as mean percentage of survival of at least three independent experiments.

Several studies on plant extracts containing polyphenols and/or saponins report a correlation between stress resistance and lifespan extension [80–85]. As CV improved the oxidative stress status of *C. elegans*, in another set of experiments, we tested its effects on the lifespan of wild-type worms. The extract significantly induced lifespan extension at all concentrations tested (Figure 7(a)) (Table 4). At 200 *μ*g/mL, maximum extension was observed, CV increased the median survival of wild-type worms from 23 to 27 d (17.29%), in comparison to the untreated control (*p* < 0.0001). Taking the previously mentioned studies into consideration, it is reasonable to assume that CV prolongs the lifespan of *C. elegans* as a result of its overall antioxidant activity.

To clarify the role of DAF-16 and SKN-1 in the CV-mediated lifespan extension, we investigated the effect of 200 *μ*g/mL CV on the lifespan of mutant worms for both transcription factors. CV failed to extend the lifespan of both mutants (Figures 7(b) and 7(c)) (Table 4). Based on these results, it may be inferred that CV prolongs *C. elegans* lifespan in a DAF-16 and SKN-1 dependent manner.

Whereas the vast majority of studies confirm the connection between increased stress tolerance and prolonged lifespan, a growing body of evidence demonstrates that defense against oxidative stress *per se* does not fully explain the mechanisms by which antioxidants extend longevity [86]. In part, our study is in agreement with this evidence. Firstly, CV treatment did not extend the lifespan in *daf-16* and *skn-1* mutants, but significantly improved their oxidative stress status reducing the levels of ROS and increasing the survival to a lethal dose of juglone. Moreover, enhanced stress resistance and lifespan extension most likely occurred by different mechanisms of action. Stress resistance was apparently not strictly dependent on DAF-16 and SKN-1, although it involved the activation of DAF-16 and the upregulation of its direct target, *sod-3.* On the contrary, CV-mediated lifespan extension required DAF-16 and SKN-1, confirming the involvement of IIS signaling in the observed effects.

#### 3.3.4. Feeding Behavior, Development, and Reproduction

**Figure 8:**
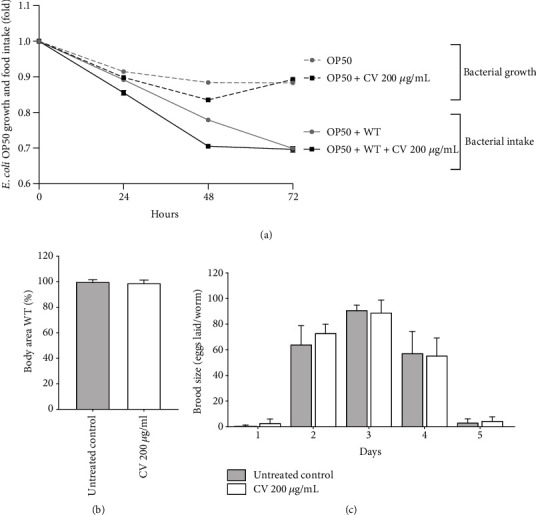
Impact of *Caryocar villosum* fruit shells (CV) on the feeding behavior, development, and reproduction of wild-type worms. (a) *E. coli* OP50 growth and food intake by means of bacterial clearance. (b) Body area of wild-type worms on the first day of adulthood. (c) Brood size. Data are representative of three independent experiments. Bacterial growth and intake are presented as fold calculated from the absorbance at day 0. Body area is expressed as percentage compared to the untreated control (mean ± SEM). Brood size represents the number of eggs laid per worm during the reproductive period (mean ± SEM).

Dietary restriction is one of the best characterized interventions to extend lifespan and stress resistance in *C. elegans* [87]. CV treatment at the highest concentration tested did not affect bacterial growth neither influenced the feeding behavior of worms, assessed by means of bacterial clearance. The worms treated with 200 *μ*g/mL CV showed a similar food intake pattern of that observed among untreated worms (Figure 8(a)). This result prompts us to rule out dietary restriction as the mechanism through which CV enhances stress resistance and prolongs lifespan in *C. elegans*.

According to the disposable soma theory, throughout the aging process, the organisms distribute their metabolic resources among various physiological processes in an effort to grow, survive, and reproduce [88]. If higher energy is put into lifespan extension (survival), energetic resources will allocate mainly into maintenance of cellular homeostasis (e.g., antioxidant defense, repair systems of nucleic acids, and proteins) resulting in compromised body growth and reproduction. CV extract is rich in polyphenols which were previously shown to extend *C. elegans* lifespan in line with the disposable soma theory [89]. To evaluate whether CV treatment affects growth and/or reproduction, we measured its effect on the worm's body area and brood size. CV at 200 *μ*g/mL did not affect the body size of wild-type worms nor the daily number of eggs laid per worm, in comparison to untreated worms (Figures 8(b) and 8(c)). Altogether, these results apparently exclude disposable soma as an explanation for the ability of CV to promote longevity in *C. elegans*.

EGCG, a polyphenol with a gallic acid moiety in its structure, has been consistently found to improve stress resistance and to prolong lifespan in *C. elegans* [62, 90–92]. Due to this background, it was employed as a positive control in this study. EGCG and CV revealed similar results; both reduced the intracellular levels of ROS rescued the worms from a lethal dose of juglone, activated DAF-16, down-regulated stress-responsive genes (*hsp-16.2* and *gst-4*), and extended lifespan in a DAF-16- and SKN-1-dependent manner.

To the best of our knowledge, from the polyphenols tentatively identified in CV, only gallic acid and ellagic acid were previously investigated in *C. elegans*, with regard to their putative antioxidant and lifespan-promoting effects. In a study comparing the mechanisms by which several polyphenols extend lifespan, Saul et al. [89] demonstrated that gallic acid (100-300 *μ*M) and ellagic acid (50 *μ*M) were able to significantly extend the lifespan of wild-type worms fed with living bacteria (as performed in the present study) but failed to increase survival under oxidative stress. At higher concentrations, gallic acid was devoid of toxicity, while ellagic acid exhibited toxic effects, reducing the lifespan of the worms under basal and oxidative stress conditions. According to the authors, the lifespan-promoting activity of gallic and ellagic acid does not seem to be correlated with their antioxidant properties. Apparently, ellagic acid might not only act through a hormetic mechanism but also as a caloric restriction mimetic, as it affects *C. elegans* feeding behavior through a chemorepellent activity. This effect was not observed for CV extract. Similarly to our results, the worm's growth and reproduction were not significantly affected, showing that gallic and ellagic acid, unlike other polyphenols, do not act in line with the disposable soma theory.

In two other studies, ellagic acid, at the same concentration previously tested, failed to extend the lifespan of wild-type worms [93, 94]. Ryu et al. [93] additionally showed the capacity of urolithin A, a digestive metabolite of ellagic acid, to sharply extend the lifespan of *C. elegans* (up to 45.4%) and to regulate mitochondrial function by mitophagy. These observations suggest that future studies on the digestive metabolites of hydrolysable tannins, rather than on their metabolic precursors, could provide important insights into the antioxidant and antiaging potential of tannin-rich plant extracts for human health.

Regarding the saponin content, none of the triterpenoid saponins identified in CV have yet been investigated. However, an isolated triterpenoid saponin from *Panax ginseng* [95], a steroidal saponin from *Asparagus racemosus* [96], and plant extracts particularly rich in triterpenoid saponins [97, 98] were previously reported to enhance stress resistance and to prolong lifespan in *C. elegans*. In the study of Magid et al. [50], saponins accounted for only 0.5% of the total composition of a methanol extract of piquiá shells. Since saponins are able to facilitate the transport of polar compounds across the cell membrane, if not bioactive, they may possibly contribute to enhance the bioavailability of the bioactive compounds of CV [99].

More studies are certainly needed to clarify which compounds, or combination of compounds, are responsible for the antioxidant and antiaging activities exerted by CV in *C. elegans*. Nonetheless, we hypothesize that the presence of free gallic and ellagic acids might be determinant for the bioactivities herein reported. These phenolic acids possess a low molecular weight, being thus presumably more bioavailable than hydrolysable tannins, which encompass highly polymerized compounds that have to be cleaved into phenolic acids in the intestinal tract in order to be absorbed.

## 4. Conclusions

Taken together, our results demonstrate the potential of piquiá shells dietary supplementation to promote stress resistance and lifespan extension in *C. elegans*. These effects implicated distinct mechanisms of action. Improved oxidative status and increased resistance to juglone-induced oxidative stress did not strictly require DAF-16 and SKN-1, while piquiá-mediated lifespan extension was shown to be dependent on both transcription factors.

In a scenario of increasing demand for antiaging interventions, our study highlights the underestimated potential of fruit waste as a source of bioactive compounds, which may be of interest for the production of antiaging dietary supplements or other applications at an industrial scale. In the specific case of piquiá shells, further studies using vertebrate model organisms are required to demonstrate whether its shells qualify as nutraceuticals. Such studies should address the bioavailability of the extract after digestion and its safety profile, taking into particular consideration the potential toxicity of gallotannins and the dual role of saponins as antinutrients or as absorption enhancers.

## Figures and Tables

**Table 1:** Tentative identification of polyphenols from a hydroalcoholic extract of *Caryocar villosum* fruit shells by LC-MS/MS analysis (ESI -).

Peak^a^	Tentative identification	*R*_t_ (min)^b^	*λ*_max_ (nm)	[M-H^+^]^−^ (*m*/*z*)	MS/MS fragments (*m*/*z*)^c^	MS^3^ fragments (*m*/*z*)^d^	Reference
1	Chebulic acid	4.8	284	355	337		[44]
2	Monogalloyl hexoside	6.8	278	331	271, 241, 211, 193, 169, 125		[43]
3	Gallic acid	7.5	264	169	125		Standard
4	HHDP-hexoside	7.8	272	481	463, 319, 301, 275	445, 301, 275	[43]
5	Digalloyl hexoside	9.5	280	483	331, 313, 271, 241, 169	313, 271, 241, 211, 169, 125	[43]
6	Unknown	10.2	269	355	337, 311, 293, 267, 205	293, 267, 223, 205, 179, 149	—
7	Unknown	12.0	273	353	334, 308, 265, 221, 177		—
8	Valoneic acid dilactone	15.1	254; 373	469	425	425, 407, 301, 300, 299	[45]
9	Galloyl-valoneoyl-glucoside isomer	18.1	266	801	757, 756, 633, 631, 613		[46]
10	Brevifolin carboxylic acid	18.8	276; 353	291	247		[53]
11	Unknown	19.5	273	1083	935		—
12	Galloyl-HHDP-hexoside (corilagin)	20.3	263	633	615, 481, 463, 419, 301, 275		[43]
13	HHDP-valoneoyl-glucoside isomer	22.1	271	951	933, 907, 799, 737, 301	863, 755, 737, 633, 605, 453, 435, 301	[47]
14	Galloyl-HHDP-DHHDP-hexoside	23.2	258; 363	951	933, 915, 613, 301		[47]
15	Galloyl-valoneoyl-glucoside isomer	24.4	265	801	757	633, 632, 631, 613, 605, 603, 587, 463, 425, 301	[46]
16	Phyllanthusiin C	28.7	273	925	907, 633, 615, 605, 453, 435, 301, 273		[48]
17	Phyllanthusiin B	29.9	272	969	951, 925, 907, 895, 851, 755, 633, 301		[48]
18	Chebulagic acid	31.9	272	953	935, 909, 801, 783, 757, 633, 481, 463, 319, 301, 275		[44, 54]
19	Digalloyl-HHDP-glucose	32.7	258; 360	785	633, 615, 589, 463, 419, 301, 275, 249		[47, 49]
20	HHDP-valoneoyl-glucoside isomer	33.2	271	951	933, 907, 836, 737, 435, 301		[47]
21	Unknown	33.4	252; 362	894	875, 806, 725, 653, 506, 447, 300, 293		—
22	Ellagic acid	33.6	252; 364	301	301, 257, 229, 185		[43]
23	HDDP-decarboxy-valoneoyl-glucoside	34.8	272; 353	907	755, 737, 633, 605, 587, 453, 435, 291, 273		—
24	Unknown	36.2	273	939	785		—
25	Unknown	37.5	272	917	747, 721, 615, 445, 301		—
26	HHDP-valoneoyl-glucoside isomer	37.7	271	951	907, 737, 649, 615, 587, 479, 435, 335, 301		[47]

^a^Peak numbers refer to the peaks presented in Figure 1. ^b^*R*_t_: retention time in TIC. ^c^The ion shown in bold face was the precursor for MS^3^ fragmentation. ^d^The MS^3^ spectra were obtained by MS/MS of ions of the source fragmentation. HHDP: hexahydroxydiphenoyl; DHHDP: dehydroxyhexahydroxydiphenoyl.

**Table 2:** Tentative identification of triterpenoid saponins from a hydroalcoholic extract of *Caryocar villosum* fruit shells by LC-MS/MS analysis (ESI +).

Peak^a^	Tentative identification	*R*_t_ (min)^b^	[M+H^+^]^+^ (*m*/*z*)	Source fragmentation	MS/MS fragments of [M + H^+^]^+^	Reference
27	Caryocaroside III-16	45.5	1431	1269, 1137, 975, 813, 651	1431, 1412, 1142, 957, 929, 826, 453	[50]
28	Caryocaroside III-14	45.9	1269	1107, 975, 813, 651	1251, 1240, 1074, 743	[50]
29	Caryocaroside III-13	46.1	1137	975, 813, 651	1138, 939, 691, 530	[50]
30	Caryocaroside III-12	46.4	1107	945, 813, 651	704, 404, 321	[50]
31	Caryocaroside III-2/III-3	46.7	975	813, 651	956, 842, 806, 738, 674	[51]
32	Caryocaroside II-16	47.2	1415	1253, 1121, 959, 797	1418, 1404, 1370, 1356, 1312, 1275, 1235, 1021, 727	[50]
33	Unknown caryocaroside	47.8	1253	1091, 959, 797	1253, 1009	—
34	Caryocaroside II-13	48.1	1121	959, 797, 635	1122, 583	[50]
35	Caryocaroside II-12	48.7	1091	929, 797, 635	1074, 833	[50]
36	Caryocaroside II-2/II-3	49.2	959	797, 635	930, 856, 831, 734	[51]
37	Caryocaroside IV-21	49.9	1429	1267, 1135, 973, 811, 649	1427, 1414, 1376, 1370, 1326, 1267, 1134, 1115, 1069, 869	[50]
38	Unknown caryocaroside	50.4	1135	973, 811, 649		—
39	Unknown caryocaroside	50.7	1267	1105, 973, 811, 649	1239, 1071, 1000	—
40	Unknown caryocaroside	51.2	1135	973, 797, 635		—
41	Caryocaroside IV-17	51.5	1105	943, 811, 649		[50]
42	Caryocaroside II-9/IV-9/II-11/IV-11/III-22	52.9	973	811	974, 928, 852, 786, 762, 657	[51, 52]
43	Caryocaroside IV-7	58.8	811	649		[52]

^a^Peak numbers refer to the peaks presented in Figure 1. ^b^*R*_t_: retention time in TIC.

**Table 3:** *In vitro* antioxidant activity of *Caryocar villosum* shells extract (CV).

Sample	DPPH IC_50_ (*μ*g/mL)	TEAC/ABTS (mM trolox/mg sample)
CV	3.01 ± 0.10	4.76 ± 0.14
Ascorbic acid	3.06 ± 0.14	5.73 ± 0.42
Gallic acid	1.12 ± 0.05	13.27 ± 0.82

DPPH: 2,2-diphenyl-1-picrylhydrazyl; TEAC: trolox equivalent antioxidant capacity; ABTS: 2,2'-azino-bis(3-ethylbenzothiazoline-6-sulfonic acid).

**Table 4:** Mean and median lifespan of wild-type (N2) and mutant worms for *daf-16* (CF1038) and *skn-1* (EU1) treated with *Caryocar villosum* fruit shells (CV).

Strain	Treatment (*μ*g/mL)	Mean lifespan ± SE (days)	Median lifespan ± SE (days)	*N*	*p* value^a^
N2	Untreated control	21.77 ± 0.29	23.00 ± 0.29	501	—
	EGCG 50	23.75 ± 0.30	25.00 ± 0.27	470	<0.0001
	CV 50	23.92 ± 0.30	25.00 ± 0.24	488	<0.0001
	CV 100	24.17 ± 0.30	25.00 ± 0.27	437	<0.0001
	CV 200	24.68 ± 0.34	27.00 ± 0.28	449	<0.0001

CF1038	Untreated control	15.46 ± 0.27	17.00 ± 0.25	339	—
	EGCG 50	14.75 ± 0.30	17.00 ± 0.49	334	0.63
	CV 200	15.61 ± 0.30	17.00 ± 0.30	304	0.27

EU1	Untreated control	13.88 ± 0.20	15.00 ± 0.27	409	—
	EGCG 50	14.08 ± 0.19	15.00 ± 0.25	415	0.54
	CV 200	13.93 ± 0.12	15.00 ± 0.33	385	0.69

^a^ *p* values (treatment vs. untreated control) were determined by the log-rank test.

## Data Availability

All data used to support the findings of this study are available from the corresponding author upon request.

## Supplementary Materials

Supplementary Materials Figure S1: representative fluorescence images of N2, CF1038, EU1, and VC475 worms analyzed for the quantification of ROS accumulation. Figure S2: representative fluorescence images of CF1553 worms analyzed for the quantification of *sod-3*::GFP levels under basal stress conditions. Figure S3: representative fluorescence images of CF1553 worms analyzed for the quantification of *sod-3*::GFP levels under juglone-induced oxidative stress. Figure S4: representative fluorescence images of CL2166 worms analyzed for the quantification of *gst-4*::GFP levels under juglone-induced oxidative stress. Figure S5: representative fluorescence images of TJ375 worms analyzed for the quantification of *hsp-16*::GFP levels under juglone-induced oxidative stress.
