# Supplementary material for: Piquiá Shells (Caryocar villosum): A Fruit by-Product with Antioxidant and Antiaging Properties in Caenorhabditis elegans
Source: Oxid Med Cell Longev. 2020 Aug 25;2020:7590707. doi: 10.1155/2020/7590707 (PMC7468659; doi:10.1155/2020/7590707)
Supplement: Supplementary Materials — Figure S1: representative fluorescence images of N2, CF1038, EU1, and VC475 worms analyzed for the quantification of ROS accumulation. Figure S2: representative fluorescence images of CF1553 worms analyzed for the quantification of sod-3::GFP levels under basal stress conditions. Figure S3: representative fluorescence images of CF1553 worms analyzed for the quantification of sod-3::GFP levels under juglone-induced oxidative stress. Figure S4: representative fluorescence images of CL2166 worms analyzed for the quantification of gst-4::GFP levels under juglone-induced oxidative stress. Figure S5: representative fluorescence images of TJ375 worms analyzed for the quantification of hsp-16::GFP levels under juglone-induced oxidative stress. [file 7590707.f1.pdf]

Supplementary materials

Piquiá shells (*Caryocar villosum*): a fruit by-product with antioxidant and anti-aging properties in *Caenorhabditis elegans*

Mariana Roxo[a], Herbenya Peixoto[a], Pille Wetterauer[a], Emerson Lima[b], Michael Wink[a*]

[a]Institute of Pharmacy and Molecular Biotechnology, Heidelberg University, Im Neuenheimer Feld 364, 69120 Heidelberg, Germany

[b]Faculty of Pharmaceutical Sciences, Federal University of Amazonas (UFAM), General Rodrigo 6200, 69077-000 Manaus, Brazil

[*]Correspondence should be addressed to Michael Wink; wink@uni-heidelberg.de

Supplementary figures

Representative fluorescence images of the worms analyzed for the measurement of intracellular ROS levels by DCF assay (section 3.3.1) and for the quantification of *sod-3*, *gst-4* and *hsp-16.2* expression under basal stress conditions and/or juglone induced oxidative stress (section 3.3.2).

All fluorescence images (λ_{ex} 488 nm and λ_{em} 540) were acquired at constant exposure time for each experiment, using a Keyence Biorevo BZ-9000 fluorescence microscope (Keyence Deutschland GmbH, Neu-Isenburg, Germany). The fluorescence intensity was densitometrically determined in mean pixels using ImageJ version 1.52 (National Institutes of Health, Bethesda, MD, USA), an open source image processing and analysis software.

ROS levels

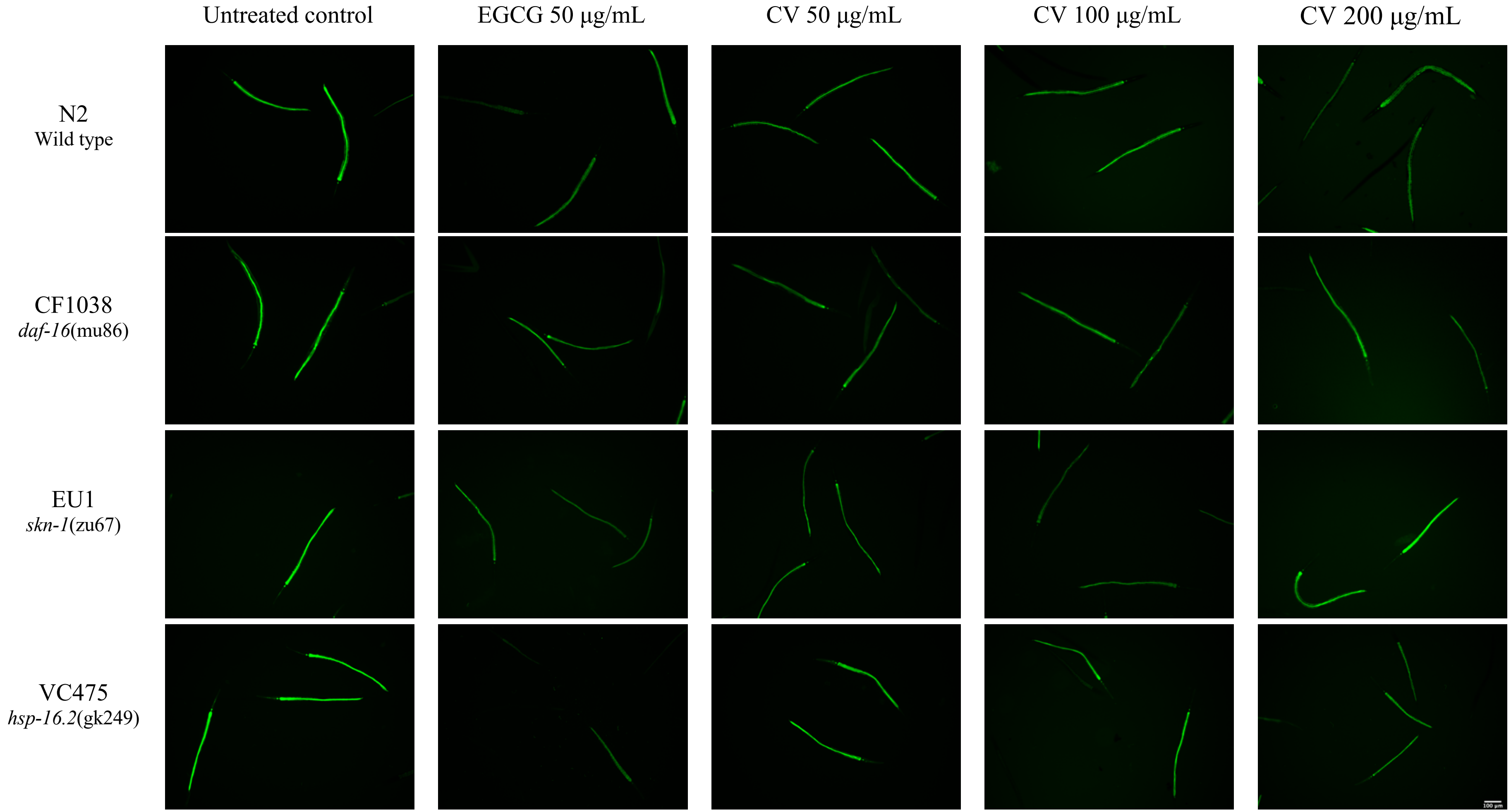

Supplementary Figure S1. Representative fluorescence images of N2, CF1038, EU1 and VC475 worms analyzed for the quantification of ROS accumulation.

Expression of stress resistance related genes

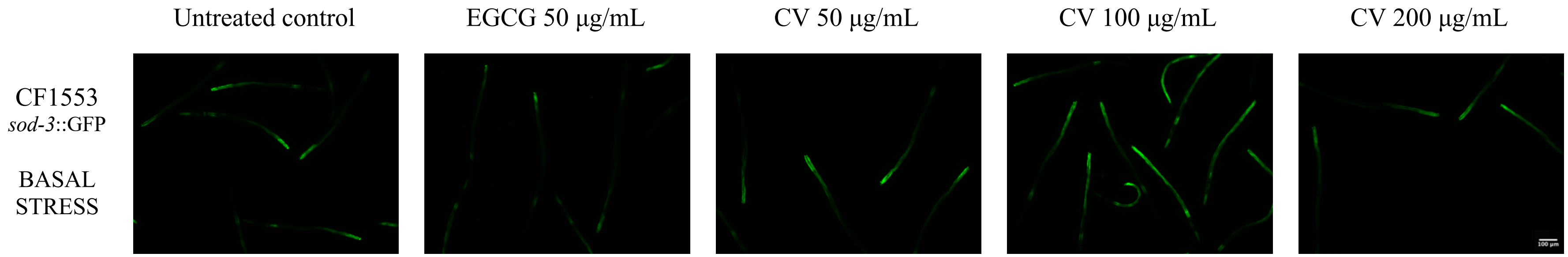

Supplementary Figure S2. Representative fluorescence images of CF1553 worms analyzed for the quantification of *sod-3*::GFP levels under basal stress conditions.

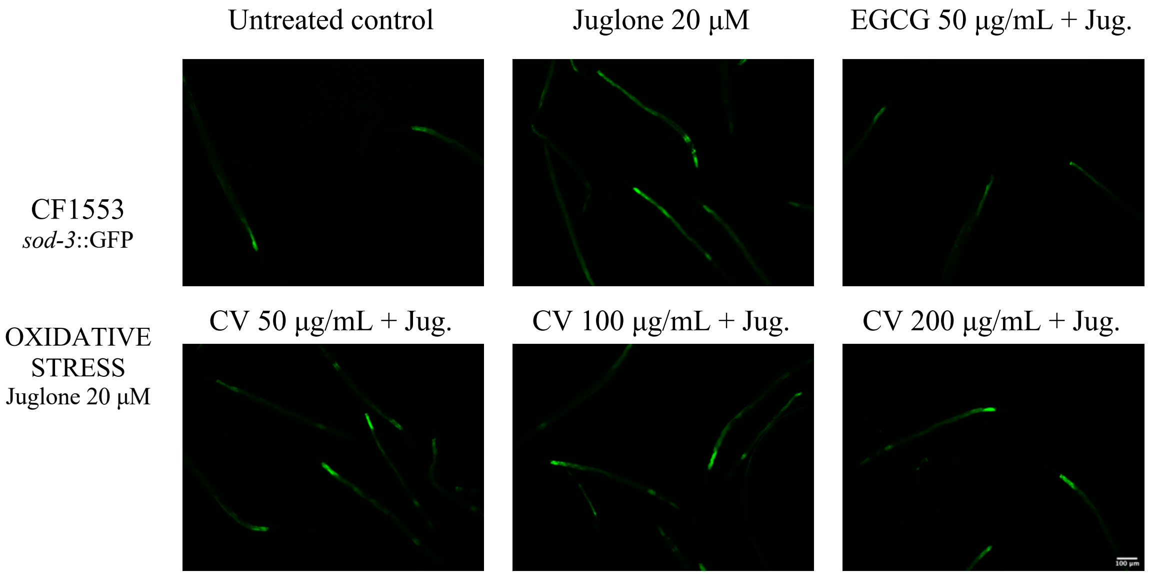

Supplementary Figure S3. Representative fluorescence images of CF1553 worms analyzed for the quantification of *sod-3*::GFP levels under juglone-induced oxidative stress.

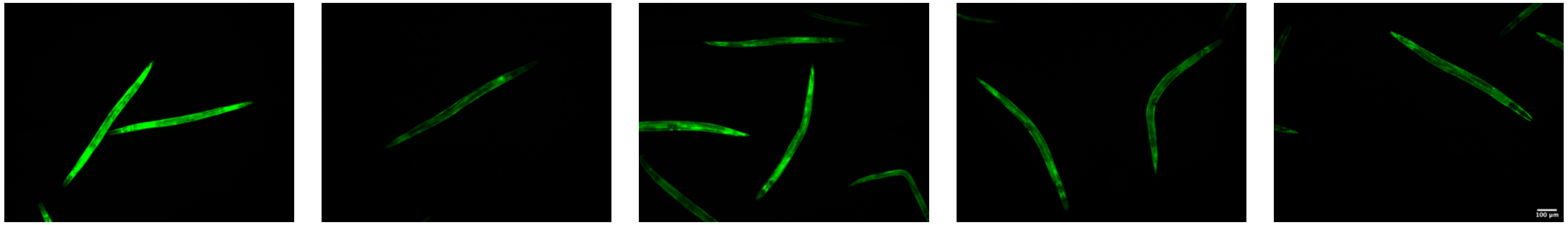

Supplementary Figure S4. Representative fluorescence images of CL2166 worms analyzed for the quantification of *gst-4*::GFP levels under juglone-induced oxidative stress.

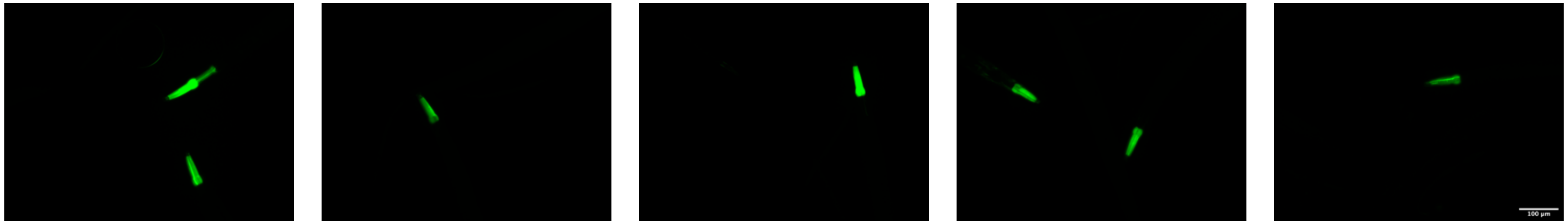

Supplementary Figure S5. Representative fluorescence images of TJ375 worms analyzed for the quantification of *hsp-16.2*::GFP levels under juglone-induced oxidative stress.
